# Effects of naftopidil on double-J stent-related discomfort: a multicenter, randomized, double-blinded, placebo-controlled study

**DOI:** 10.1038/s41598-017-04505-y

**Published:** 2017-06-23

**Authors:** Jong Jin Oh, Sangchul Lee, Sung Yong Cho, Sang Wook Lee, Min Chul Cho, Woong Na, Ju Hyeon Park, Seung Bae Lee, Soyeon Ahh, Chang Wook Jeong

**Affiliations:** 1 Department of Urology, Seoul National University College of Medicine, Seoul National University Bundang Hospital, Seongnam, Korea; 2 Department of Urology, Seoul Metropolitan Government Seoul National University Boramae Medical Center, Seoul, Korea; 3 Department of Urology, Kangwon National University School of Medicine, Chuncheon, Korea; 4 Department of Urology, National Medical Center, Seoul, Korea; 5 Department of Urology, Sheikh Khalifa Specialty Hospital, Ras Al Khaimah, UAE; 6 Division of Statistics, Medical Research Collaborating Center, Seoul National University Bundang Hospital, Seongnam, Korea; 7 Department of Urology, Seoul National University Hospital, Seoul, Korea

## Abstract

To evaluate the effect of naftopidil 75 mg once daily for ureteral double-J (DJ) stent-related discomfort after a ureteroscopic procedure using a multicenter, randomized, double-blinded, placebo-controlled study. 100 patients with indwelled retrograde DJ ureteral stents after ureteroscopic stone removal or retrograde intrarenal surgery (RIRS) were randomized 1:1 to receive either placebo or naftopidil during the stenting period. At the time of stent removal, the Ureteral Stent Symptom Questionnaire (USSQ), the International Prostate Symptom Score and the total amount of used analgesics were reported. Of the 92 patients who completed the study, 49 patients were enrolled in the placebo group, and 43 patients in the naftopidil group. USSQ urinary symptom scores (30.90 vs. 29.23, p = 0.299) and USSQ body pain scores (22.28 vs. 19.58, respectively, p = 0.286) were lower in the naftopidil group than in the placebo group, but the difference was not significant. Multivariate analysis showed that the use of a ureteral access sheath during RIRS was the only significant predictor of postoperative DJ-related pain (OR = 2.736, p = 0.031). The use of naftopidil once daily did not significantly reduce DJ ureteral stent-related discomfort. Larger-scaled prospective studies should be conducted to evaluate the effects of naftopidil on DJ stent-related symptoms and surgeries.

## Introduction

Ureteral stenting has been an established urological procedure for maintaining the patency of ureters since 1967^1–3^. Although ureteral stents enable passive ureteral dilation and divert the flow of urine to the bladder, these stents have been associated with significant morbidity and have had negative effects on quality of life^4^. Previous studies have reported high incidences of urination frequency (50–60%), urgency (57–60%), dysuria (40%), incomplete emptying (76%), flank (19–32%) and suprapubic pain (30%), and hematuria (25%)^5–7^. Other reports have shown that stent-related urinary symptoms and pain have resulted in reduced quality of life (QoL) in up to 80% of patients^4^.

Many studies have sought to reduce ureteral stent-related discomfort by investigating the effect of α-blockers, which have shown some beneficial effects in reducing stent-related symptoms^8–16^. However, these studies used tamsulosin or alfuzosin and not naftopidil. α1-adrenergic receptors are known to be present throughout the ureter, and the blockage of α1-adrenergic receptors inhibits basal smooth tone and hyperperistaltic frequency^17^. Among α1-adrenergic receptors, α1D-adrenoceptors are prevalent throughout the distal ureter to the detrusor muscle^18^. Because naftopidil has greater selectivity for the α1D-adrenoceptor^19^, there is a theoretical advantage in using naftopidil to reduce bladder irritation symptoms or pain due to ureteral stents.

To obtain higher-level evidence showing the effects of naftopidil on reducing double-J (DJ) ureteral stent-related discomfort, we conducted a multicenter, double-blind, randomized, controlled trial (RCT). To evaluate the effect of naftopidil on improving symptoms and quality of life in patients with indwelling ureteral stents, we used the validated Ureteral Stent Symptom Questionnaire (USSQ).

## Materials and Methods

### Ethics statement

This study was a multicenter double-blinded RCT from 6 institutions. After obtaining institutional review board approvals from each institution, 100 patients were selected. This study design for use of naftopidil for patients with a ureter stone was approved by the Ministry of Food and Drug Safety (the approval number 12449, June 4, 2013). This study is registered at www.clinicaltrials.gov under registry number- NCT01959074 (September 24, 2013). The methods were performed in accordance with approved guidelines.

### Study participants

The patients were selected based on the following inclusion criteria: patients ≥18 years age who underwent unilateral retrograde ureteral DJ stent placement only after ureteroscopic surgery (URS) and retrograde intrarenal surgery (RIRS). The following patients were excluded: (1) patients who underwent percutaneous nephrolithotomy, open ureteral surgery or laparoscopic ureteral surgery, including ureterolithotomy; (2) patients with persistent ureteral stones after ureterolithotomy; (3) patients with a febrile urinary tract infection (UTI); (4) patients who were pregnant or breast feeding at the time of surgery; (5) patients with a single kidney; (6) patients with hypersensitivity to naftopidil; (7) patients using α -blockers, calcium channel blockers, anti-muscarinic medication or corticosteroids within 4 weeks; (8) patients with moderate or severe cardiovascular or cerebrovascular disease; (9) patients with moderate hepatic dysfunction (i.e., liver function test results >2× normal); (10) patients with a prior history of pelvic surgery or irradiation; (11) patients with a prior history of transurethral surgery; (12) patients with significant active medical illnesses precluding protocol treatment and 13) patients with chronic kidney disease stage IV (glomerular filtration rate < 30 ml/min/1.73 m^2^). The patients’ Informed consents were received before the study (enrollment and publication of results).

### Study design

From Jan 2014 to May 2015, eligible patients were randomly and equally divided into a naftopidil 75 mg once daily group and a placebo group. Randomization was based on a computer-generated random table at a central institution. The appropriate drugs and placebos were delivered to the allocation centers. At each center, sequentially numbered boxes containing the complete treatment for each patient were delivered to the investigator by the pharmacist following the randomized schedule. All patients, attending urologists, and investigators were not aware of the assignments throughout the study period.

Sample size calculations were performed based on a previous meta-analysis investigating the effect of α-blockers in reducing ureteral stent-related symptoms^20^. The USSQ urinary symptom score and the USSQ body pain score were 20.0 (with a standard deviation [SD] of 5.6) and 11.9 (SD 10.1) in a group using an α-blocker and 28.4 (SD 6.2) and 19.1 (SD 10.9) in a group receiving placebo, respectively. A log-rank test with a two-sided significance level of 0.025 had 80% power to detect differences between the groups, with a sample size of 45 in each group. Fifty patients per group were randomized, which allowed for a maximum dropout rate of 15%.

### Intervention

The patients were directed to take the study medication from the day after stent placement to the day of stent removal. The maximal stent indwelling period was 15 days after ureteral surgery (duration: 5–15 days). For acute intractable pain after ureteral stent indwelling, only aceclofenac 100 mg, a non-steroidal anti-inflammatory drug, was provided on demand. Patients were asked to keep a diary recording analgesic usage amounts, periods of absence from work, and the presences and types of side effects believed to be caused by the medication. Follow-up exams were performed on the day of stent removal. All patients completed the USSQ and IPSS scoring and reported a final analgesic usage amount. All patients completed IPSS preoperatively and the day of stent removal. The USSQ questionnaires were obtained in all patients on the day of stent removal. All questionnaires were collected by operating surgeon. The same double-J ureteral stent (Percuflex Plus, Boston Scientific, Natick, MA) was used in all patients. The stent diameter was 6 Fr, and the length was dependent on the somatometric characteristics of each patient. The stent was inserted under fluoroscopic guidance in all cases. Stent position after surgery was confirmed by radiography.

### Endpoint

The primary endpoints were the USSQ urinary symptom score and USSQ body pain score. The secondary endpoints were the USSQ general health score, USSQ work performance score, USSQ sexual matter score, IPSS score and QoL score, as well as total analgesic use.

### Statistical analysis

Statistical analyses were performed using SPSS version 18.0 on an intent-to-treat basis. Patients with major deviations from the inclusion criteria were excluded from the analysis. Stone size was measured preoperatively at maximal length. And in multiple stone, we calculated as the sum of the largest axis of each stone on computed tomography (CT). Fisher’s exact test was used to compare nominal variables, and the Mann–Whitney U test was used to compare continuous variables between the two treatment arms before ureteral stenting. To compare outcomes after ureteral stent removal, a Chi-square and non-parametric Wilcoxon 2-sample t-test were used. In the post-hoc analyses, multivariate logistic regression was used to predict the effective factors for higher USSQ body pain scores. Multivariate logistic regression analysis was also used to find the predictor of postoperative stent-related pain. A two-sided p < 0.05 was considered to be statistically significant.

## Results

### Study population

**Figure 1:**
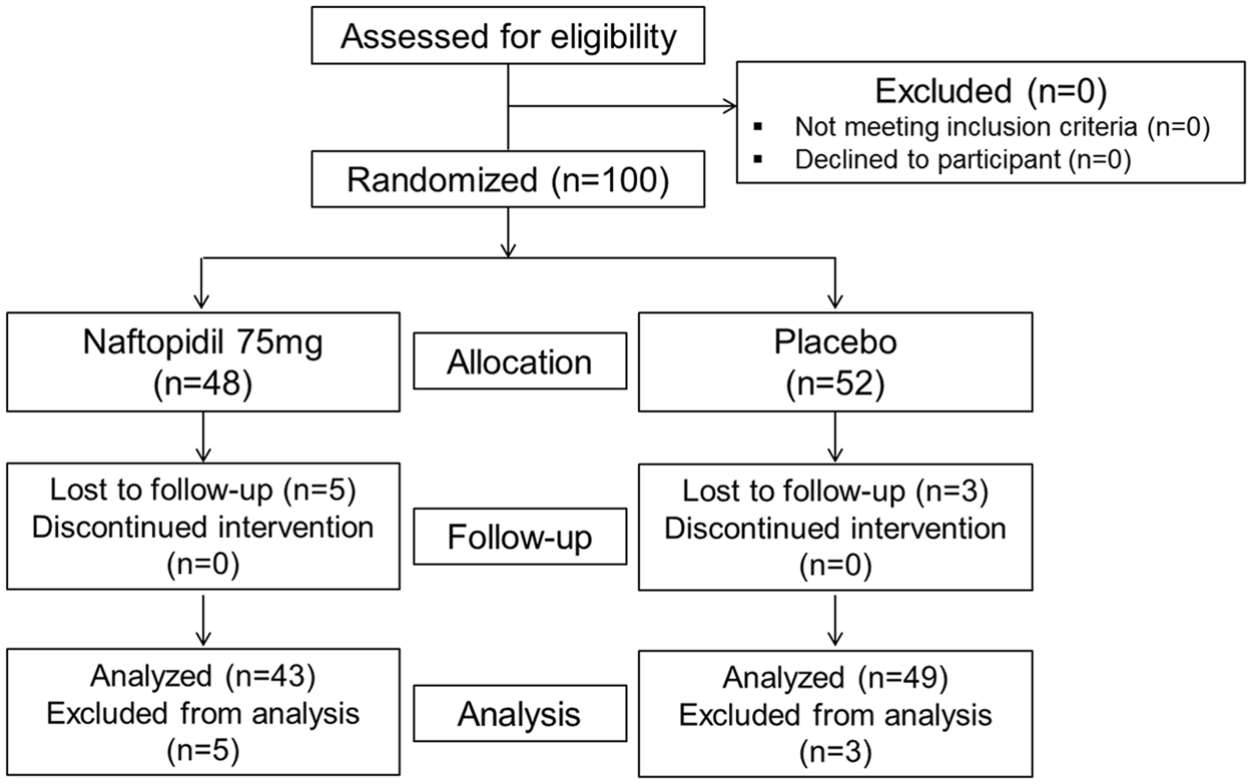
CONSORT diagram of this study.

Figure 1 shows the CONSORT diagram of this study. A total of 100 patients from 6 tertiary institutions were randomly assigned to the treatment and placebo arms. Overall, eight patients were excluded from the final analysis because of 1 major deviation from the inclusion criteria and 7 non-completed questionnaires.

Drug compliance was check by patients’ reports and envelope. There are 6 drug related complication. The abdominal discomfort was reported 3 patients in control group and 1 patient in naftopidil group. One patient in control group reported diarrhea, one patient in naftopidil group reported chest discomfort. All reported symptoms were recorded unrelated to drugs and there was no significant difference between two groups.

The patient characteristics of each arm are shown in Table 1. Mean age and body mass index (BMI) and stone size, location and position were not different between two groups. The preoperative IPSS was 9.33 in the control group and 9.35 in the naftopidil group (i.e., no significant difference). In the control group, 22 patients (44.9%) underwent URS using a rigid ureteroscope, and 22 patients (44.9%) underwent RIRS using a ureteral access sheath (UAS). In the naftopidil group, 16 patients (37.2%) underwent URS with a rigid ureteroscope, and 25 patients (58.1%) underwent RIRS using a UAS.

**Table 1:** Baseline characteristics.

	Placebo	Naftopidil 75 mg	p-value
Number of patients	49	43	
Age, mean (SD) (years)	52.35 ± 13.43	50.88 ± 13.78	0.608
Sex, no (%)			0.048
Male	23 (46.9)	29 (67.4)	
Female	26 (53.1)	14 (32.6)	
Body mass index, mean (SD) (kg/m^2^)	24.07 ± 3.56	24.61 ± 4.98	0.550
Stone size, mean (SD) (mm)	9.33 ± 6.27	11.25 ± 6.09	0.142
Stone location (%)			0.784
Right	21 (42.9)	16 (37.2)	
Left	25 (51.0)	25 (58.1)	
Bilateral	3 (6.1)	2 (4.7)	
Stone position (%)			0.766
Kidney	14	18	
Upper ureter	8	7	
Mid ureter	6	6	
Low ureter	12	6	
Multiple site	9	6	
Preoperative IPSS	9.33 ± 6.84	9.35 ± 6.83	0.988
Preoperative IPSSstorage	4.27 ± 3.13	4.09 ± 3.11	0.792
Preoperative IPSSvoiding	5.06 ± 4.67	5.26 ± 4.71	0.843
Preoperative QoL	2.82 ± 1.70	2.35 ± 1.51	0.173
Surgery type (%)			0.476
URS with rigid ureteroscope	22 (44.9)	16 (37.2)	
URS with flexbile ureteroscope	4 (8.2)	2 (4.7)	
RIRS without UAS	1 (2.0)	0 (0.0)	
RIRS with UAS	22 (44.9)	25 (58.1)	
Presence of preoperative Double-J stenting	13 (26.5)	13 (30.2)	0.694
Duration of double-J ureteral stent	12.09 ± 5.59	10.81 ± 3.90	0.220

SD, standard deviation; IPSS, international prostate symptom score; URS, ureteroscopy; RIRS, retrograde intrarenal surgery; UAS, ureteral access sheath.

### Primary endpoint

The overall results are listed in Table 2, which shows postoperative symptom scores based on the USSQ. The USSQ urinary symptom score was 30.90 in the placebo group and 29.23 in the naftopidil group (not significantly different, p = 0.299). Among the USSQ urinary symptom scores, U2 and U3, which represent nocturia and urgency, respectively, were lower in the naftopidil group than in the control group (p = 0.031 and p = 0.030, respectively). Other sub points of the USSQ urinary symptoms score were not different between the two groups. The USSQ body pain score was 22.28 in the placebo group and 19.58 in the naftopidil group (p = 0.286). The prevalence of inguinal pain was lower in the naftopidil group (14.0%) than in the placebo group (34.7%; p = 0.026). Other USSQ body pain scores were similar between the two groups.

**Table 2:** Overall study results of primary end point and secondary end point.

	Placebo	Naftopidil 75 mg	p-value
Primary end point			
USSQ urinary symptom score	30.90 ± 7.60	29.23 ± 7.66	0.299
U1-daytime frequency	3.29 ± 1.19	3.26 ± 1.18	0.904
U2-nocturia	2.78 ± 1.14	2.30 ± 0.89	0.031
U3-urgency	2.37 ± 1.33	1.86 ± 0.83	0.030
U4-urge incontinence	1.63 ± 1.01	1.63 ± 0.90	0.981
U5-urge incontinence	1.39 ± 0.89	1.33 ± 0.72	0.714
U6-residual urine sense	2.84 ± 1.46	2.63 ± 1.22	0.462
U7-dysuria	2.49 ± 1.33	2.56 ± 1.42	0.812
U8-hematuria rate	3.24 ± 1.42	3.23 ± 1.40	0.967
U9-hematuria amount	2.39 ± 1.34	2.33 ± 0.84	0.751
U10-total symptom	2.92 ± 1.17	2.70 ± 1.28	0.390
U11-QoL about stent	5.57 ± 1.34	5.42 ± 1.30	0.583
USSQ body pain score	22.28 ± 12.26	19.58 ± 11.75	0.286
P1- presence of pain (%)	43 (87.8)	36 (83.7)	0.398
P2-presence of flank pain (%)	9 (18.4)	8 (18.6)	0.535
P3-VAS of flank pain	1.50 ± 2.42	2.04 ± 3.02	0.594
P2-presence of inguinal pain (%)	17 (34.7)	6 (14.0)	0.026
P3-VAS of inguinal pain	2.65 ± 2.74	1.54 ± 2.67	0.264
P2-presence of suprapubic pain (%)	23 (46.9)	17 (39.5)	0.404
P3-VAS of suprapubic pain	3.43 ± 3.25	4.33 ± 3.64	0.442
P2-presence of back pain (%)	13 (26.5)	12 (27.9)	0.457
P3-VAS of back pain	2.76 ± 2.97	3.07 ± 3.71	0.800
P2-presence of genitalia pain (%)	7 (14.3)	10 (23.3)	0.220
P3-VAS of genitalia pain	1.13 ± 2.10	3.40 ± 3.85	0.274
P4-physical activity	3.13 ± 1.31	3.13 ± 1.28	0.995
P5-sleep difficulty	2.27 ± 1.34	2.16 ± 1.18	0.698
P6-pain during voiding	3.31 ± 1.36	3.05 ± 1.38	0.394
P7-flank pain during voiding (%)	17 (34.7)	15 (34.9)	0.527
P8-analgesics	2.69 ± 1.43	2.34 ± 1.17	0.235
P9-QoL	2.96 ± 1.17	2.97 ± 1.26	0.946
USSQ general health score	15.16 ± 5.47	14.84 ± 5.67	0.780
USSQ work performance score	3.67 ± 4.25	4.70 ± 4.99	0.291
**USSQ sexual matter score**			
Stent related sexual abstinence (%)	12 (24.5)	5 (11.6)	0.133
Postoperative IPSS score	9.17 ± 8.56	8.81 ± 9.94	0.855
Postoperative QoL score	3.36 ± 1.58	2.98 ± 2.02	0.320
total analgesics use, mean (tablet)	7.55 ± 11.92	5.46 ± 5.87	0.349

USSQ, Ureteral stent symptom questionnaire; IPSS, international prostate symptom score; QoL, quality of life; VAS, visual analogue scale.

### Secondary endpoint

As shown in Table 2, the USSQ general health score was 15.16 in the placebo group and 14.84 in the naftopidil group (p = 0.780). The USSQ work performance score was 3.67 in the placebo group and 4.70 in the naftopidil group (p = 0.291). The rate of sexual abstinence due to stent-related discomfort was 24.5% in the placebo group and 11.6% in the naftopidil group (p = 0.133). The postoperative IPSS score was relatively low in the naftopidil group. However, no significant difference was observed (p = 0.855). The total amount of analgesics used was 7.55 tablets in the control group and 5.46 tablets in the naftopidil group (p = 0.349).

### Multivariate analysis

Multivariate analysis was used to determine the predictor of postoperative DJ stent-related pain score based on the USSQ body pain results (Table 3). Based on a univariate logistic analysis to determine the predictor of a USSQ pain score above 20, the preoperative IPSS, preoperative storage symptom (IPSS) and surgery type were considered significant factors. Other factors, as shown in Table 3, were not considered significant factors. After multivariate analysis, surgery type (RIRS) was only the significant predictor of postoperative DJ-related pain (OR = 2.736, 95% CI: 1.095–6.834, p = 0.031).

**Table 3:** Multivariate logistic analysis to find the predictor of double J ureteral stent related pain based on USSQ pain score.

	Univariate analysis			Multivariate analysis		
	OR	95% CI	p-value	OR	95% CI	p-value
Naftopidil (vs control)	0.691	0.303–1.577	0.380	0.771	0.313–1.901	0.572
Age (yr)	0.983	0.953–1.014	0.276			
Preoperative IPSS	1.076	1.007–1.149	0.030	2.923	0.830–10.286	0.095
Preoperative storage symptoms	1.180	1.020–1.364	0.026			
Preoperative voiding symptoms	1.083	0.988–1.187	0.089			
Surgery type (rigid vs flexible using UAS)	2.561	1.053–6.229	0.038	2.736	1.095–6.834	0.031
Preoperative DJ stenting (yes vs no)	0.779	0.312–1.947	0.593			
Operation time (min)	1.006	0.996–1.017	0.248			
Stone diameter (mm)	1.025	0.958–1.097	0.474			
Length of DJ stent (yes vs no)	0.944	0.408–2.188	0.894			
Duration of DJ stenting (days)	1.051	0.961–1.148	0.277			
Presence of midline cross of DJ stent (yes vs no)	1.619	0.659–3.978	0.293			

USSQ, Ureteral stent symptom questionnaire; OR, odds ratio; CI, confidence interval; IPSS, international prostate symptom score; DJ, double J; UAS; ureteral access sheath.

### Subgroup analysis

Table 4 showed the odds ratio between naftopidil group and placebo group for the DJ stent related urinary symptoms and pain. In the subgroup which patients who underwent surgery using rigid URS or RIRS without UAS, naftopidil was significantly effective decreased the DJ related pain on USSQ-pain score, which was significantly lower in the naftopidil group (16.83 ± 9.79) than in the control group (20.52 ± 12.35; OR = 0.431, 95% CI (0.131–0.891), p = 0.041).

**Table 4:** Subgroup Analyses of odds Ratios for ureteral stent related urinary symtoms (USSQ urinary symptom score) and ureteral stent related pain (USSQ-body pain score) in the two Groups.

Subgroup	No. of patients	Placebo	Naftopidil	Odds ratio (95% CI)
**USSQ urinary symptom score**				
All patients	92	30.90 ± 7.60	29.23 ± 7.66	0.760 (0.334–1.729)
Age				
<50 yr	36	30.00 ± 7.22	30.07 ± 6.72	1.039 (0.275–3.919)
≥50 yr	56	31.57 ± 7.93	28.79 ± 8.20	0.647 (0.224–1.868)
Stone size				
<10 mm	52	30.39 ± 7.56	28.26 ± 6.99	0.700 (0.220–2.226)
≥10 mm	40	31.94 ± 7.82	30.00 ± 8.22	0.600 (0.165–2.180)
Stone position				
Kidney	44	32.67 ± 9.01	29.35 ± 8.48	0.385 (0.113–1.310)
Ureter	48	29.57 ± 6.19	29.10 ± 6.81	1.264 (0.395–4.043)
Preoperative IPSS				
<12	62	29.85 ± 6.97	27.79 ± 7.23	0.649 (0.235–1.790)
≥12	30	33.06 ± 8.59	32.21 ± 7.92	1.037 (0.244–4.411)
Surgery type				
RIRS with UAS	47	32.82 ± 8.75	29.84 ± 8.36	0.431 (0.131–1.419)
Others	45	29.33 ± 6.25	28.39 ± 6.71	1.082 (0.317–3.694)
Duration of ureteral stent				
<10	42	30.17 ± 7.09	27.90 ± 6.95	0.625 (0.170–2.291)
≥10	50	31.69 ± 7.45	30.43 ± 8.44	0.894 (0.290–2.757)
**USSQ body pain score**				
All patients	92	22.28 ± 12.26	19.58 ± 11.75	0.691 (0.303–1.577)
Age				
<50yr	36	25.02 ± 13.78	23.40 ± 13.03	1.039 (0.275–3.919)
≥50 yr	56	20.21 ± 10.76	17.54 ± 10.69	0.556 (0.190–1.621)
Stone size				
<10 mm	52	20.95 ± 12.95	19.84 ± 12.44	0.873 (0.279–2.728)
≥10 mm	40	25.00 ± 10.53	19.38 ± 11.44	0.429 (0.117–1.568)
Stone position				
Kidney	44	24.19 ± 12.32	21.57 ± 13.37	0.385 (0.113–1.310)
Ureter	48	20.84 ± 12.22	17.30 ± 9.37	1.030 (0.319–3.329)
Preoperative IPSS				
<12	62	21.11 ± 11.48	17.90 ± 12.88	0.495 (0.178–1.382)
≥12	30	24.69 ± 13.78	23.07 ± 8.33	1.333 (0.315–5.642)
Surgery type				
RIRS with UAS	47	24.43 ± 12.06	21.56 ± 12.80	0.850 (0.243–2.976)
Others	45	20.52 ± 12.35	16.83 ± 9.79	0.431 (0.131–0.891)
Duration of ureteral stent				
<10 days	42	21.50 ± 12.44	18.10 ± 13.22	0.500 (0.133–1.885)
≥10 days	50	22.91 ± 11.56	20.67 ± 10.39	0.894 (0.290–2.757)

USSQ, Ureteral stent symptom questionnaire; IPSS, international prostate symptom score; RIRS, retrograde intrarenal surgery; UAS, ureteral access sheath.

Table 5 shows the differences in IPSS after surgery according to medication. The total IPSS score decreased from 9.33 to 9.17 (with a mean decrease of 0.09 points) in the placebo group and from 9.35 to 8.81 (with a mean decrease of 0.53 points) in the naftopidil group (p = 0.809). Twenty-two patients (44.9%) in the control group and 14 patients (32.6%) in the naftopidil group showed increased IPSS scores at the time of DJ stent removal. Among the patients with preoperative IPSS scores lower than 12, the IPSS score increased after surgery from 5.94 to 7.80 in the control group and from 5.90 to 6.00 in the naftopidil group. Eighteen patients (51.4%) in the control group and 8 patients (25.8%) in the naftopidil group had increased IPSS scores (p = 0.030).

**Table 5:** Subgroup analysis according to international prostate symptom score.

Total patients	Placebo	Naftopidil	p-value
	N = 49	N = 43	
Preoperative IPSS score	9.33 ± 6.84	9.35 ± 6.83	0.988
Postoperative IPSS score	9.17 ± 8.56	8.81 ± 9.94	0.855
Difference IPSS score	−0.09 ± 8.23	−0.53 ± 9.40	0.809
The number of IPSS score increased (%)	22 (44.9)	14 (32.6)	0.122
**Preoperative low IPSS score patients**	**N = 35**	**N = 31**	
Preoperative IPSS score	5.94 ± 3.68	5.90 ± 3.26	0.962
Postoperative IPSS score	7.80 ± 7.74	6.00 ± 7.94	0.355
Difference IPSS score	1.94 ± 7.61	0.10 ± 8.95	0.369
The number of IPSS score increased (%)	18 (51.4)	8 (25.8)	0.030

IPSS, international prostate symptom score.

## Discussion

This double-blinded multicenter RCT demonstrated the effect of naftopidil 75 mg once daily on DJ stent-related discomfort. The overall outcomes based on the primary endpoint (i.e., USSQ urinary symptoms score and USSQ body pain score) showed no significant effects on the aforementioned symptoms. However, we found that the surgical type, especially RIRS using a UAS, was a significant factor associated with postoperative pain. Significant differences were observed between patients who were and were not subjected to RIRS with a UAS. This is the first study to use naftopidil to investigate the effects of DJ-related discomfort.

Stent-related discomfort, pain or urinary symptoms may be associated with ureteric spasm or trigonal irritation. Ureteric and local trigonal smooth muscle relaxation and reduced ureteric motility could reduce these symptoms^20, 21^. Additionally, relaxation of the bladder neck and the prostatic smooth muscle and, consequently, a reduction in voiding pressure and urinary reflux could reduce pain during voiding^12^. Through these mechanisms, the role of alpha-blockers in ureteral stent-related symptoms could be better focused (alpha-blockers relieve pain by decreasing the muscle tone of the ureter, bladder trigone and prostatic urethra by blocking alpha-adrenergic receptors, thereby reducing bladder outlet resistance and pressure during micturition).

Several RCTs have shown that alpha blockers reduce stent-related symptoms. Wang *et al*.^12^ studied tamsulosin 0.4 mg once daily in a RCT of 154 patients. Tamsulosin 0.4 mg decreased the USSQ urinary symptom score from 31.59 in the placebo group to 20.96 in the tamsulosin group (p < 0.0001) and the USSQ body pain score from 13.3 in the placebo group to 9.94 in the tamsulosin group (p = 0.04). Dellis *et al*.^22^ observed the effects of tamsulosin 0.4 mg and alfuzosin 10 mg in a 3-armed RCT of 150 patients. Both alpha blockers significantly reduced USSQ urinary symptom and body pain scores. More recently, Singh *et al*.^23^ showed that tamsulosin 0.4 mg had positive effects on urinary index and pain index scores in a small RCT (n = 60).

However, a few RCTs investigating the effects of alpha-blockers on stent-related symptoms have reported negative results. In a 104 patient RCT, Tehranchi *et al*.^24^ showed that terazosin 2 mg twice a day reduced urinary symptoms and pain. Beddingfield *et al*.^9^ showed that alfuzosin 10 mg could not effectively reduce USSQ pain scores and USSQ urinary symptoms scores. Whereas these studies provided conflicting results, conclusive meta-analyses have shown that alpha-blockers significantly reduce USSQ urinary symptoms (−6.37; p < 0.0001) and body pain index (−7.03; p = 0.0008) scores^25^ and reduce urinary symptoms (−6.76; p 0.005) and body pain (−3.55, p = 0.0004)^26^.

Recently, many studies have shown tamsulosin to effectively reduce DJ stent-related pain. The α1A- or α1D-selective blocker is effective over a wide predisposition of α1D adrenoceptors from the distal ureter to the bladder detrusor muscle^17, 18^. Naftopidil has a higher selectivity for the α1D-adrenoceptor, having approximately threefold and 17-fold higher potency for α1D than for α1 A and α1B, respectively^19^. Thus, there is a theoretical advantage in using naftopidil in studying DJ stent-related symptoms^27^.

Several studies showed their efficacy of anti-muscarinic agents or combination α blocker and anti-muscarinic drugs. El-Hahas *et al*.^28^ showed effectiveness of tamsulosin 0.4 mg and solifenacin 5 mg in relieving ureteral stents related symptoms, tamsulosin group and solifenacin group both improved the DJ stent related symptoms, however solifenacin had more effectively release the DJ stent related discomfort. Another reports from Liu *et al*.^29^ showed combination of tamsulosin and solifenacin had faster effect than monotherapy during initial period of DJ stent indwelling. However, Sivalingam *et al*.^30^ recently compared tamsulosin 0.4 mg plus eolterodine 4 mg and tamsulosin 0.4 mg alone, their results showed no significant difference in urinary symptoms, body pain and daily activity among patients who indwelled DJ stent. Therefore combination effect of α blocker and anti-muscarinic drugs should be necessary further evaluation. The study about combination of anti-muscarinic and naftopidil for the DJ stent related symptoms was not present, we should be focused these combinational effect in near future.

To our knowledge, this is the first RCT using naftopidil to decrease ureteral stent-related symptoms. Unexpectedly, we observed that naftopidil 75 mg once daily could not effectively reduce urinary symptoms or body pain based on the USSQ. This observation could be due to the use of RIRS with a UAS. As shown in our multivariate analyses, surgery type (RIRS) was the main factor predicting postoperative DJ-related pain. Because we typically use a UAS with or without ureteral dilation during RIRS and because the typical UAS outer diameter is 14 Fr or 16 Fr, these larger diameter and longer ureteral dilation times could affect urinary symptoms and body pain. In the subgroup analysis among patients who underwent rigid URS not using a UAS, naftopidil 75 mg once daily treatment significantly decreased USSQ pain scores compared with the control group. However, the subgroup analysis included only 45 patients. These pain scores may have been different if the study had focused only on men who underwent rigid URS.

This study had some limitations. First, a relatively small number of patients was sampled. Although the calculated sample size was sufficient, with a significance level of 0.025 and 80% power, eight patients were excluded from the final analysis. Second, the uneven allocation of patients to the placebo group (n = 52) and the naftopidil group (n = 48) was due to the simultaneous multicenter recruitment of patients. Finally, the surgery type was not consistent. We originally assumed that DJ-related pain was not associated with surgery type, such as RIRS. However, RIRS, which uses a large diameter UAS, may be an important factor affecting postoperative urinary and pain symptoms. Another RCT could be conducted to compare postoperative discomfort after rigid URS or RIRS.

In conclusions, the use of naftopidil 75 mg once daily among ureteroscopic surgery patients could not effectively decrease DJ-related urinary and pain symptoms. Among the patients who underwent endoscopic stone surgery without UAS, naftopidil could be helpful to reduce DJ related pain. Future RCTs could assess the impact of surgery types.
